# A Meta-Analysis of *GBA*-Related Clinical Symptoms in Parkinson's Disease

**DOI:** 10.1155/2018/3136415

**Published:** 2018-09-27

**Authors:** Yuan Zhang, Li Shu, Xun Zhou, Hongxu Pan, Qian Xu, Jifeng Guo, Beisha Tang, Qiying Sun

**Affiliations:** ^1^Department of Neurology, Xiangya Hospital, Central South University, Changsha, Hunan 410008, China; ^2^National Clinical Research Center for Geriatric Disorders, Changsha, Hunan 410078, China; ^3^Key Laboratory of Hunan Province in Neurodegenerative Disorders, Central South University, Changsha, Hunan 410008, China; ^4^Parkinson's Disease Center of Beijing Institute for Brain Disorders, Beijing 100069, China; ^5^Collaborative Innovation Center for Brain Science, Shanghai 200032, China; ^6^Collaborative Innovation Center for Genetics and Development, Shanghai 200438, China; ^7^Department of Geriatrics, Xiangya Hospital, Central South University, Changsha, Hunan 410008, China; ^8^Center for Medical Genetics, School of Life Sciences, Central South University, Changsha, Hunan 410008, China

## Abstract

**Background:**

*GBA* gene had been proved to be a crucial gene to the risk of PD. Numerous studies had discussed about the unique clinical characteristics of PD patients with *GBA* carriers (*GBA* + PD). However, there was lack of updated comprehensive analysis on the topic. In order to clarify the association between *GBA* variants and the clinical phenotypes of PD, we conducted this comprehensive meta-analysis.

**Method:**

Medline, Embase, and Cochrane were used to perform the searching. Strict selection criteria were followed in screening for new published articles or data. Revman 5.3 software was applied to perform the total statistical analysis, and funnel plots in the software were used to assess the publication biases.

**Results:**

A total of 26 articles including 931 *GBA* + PD and 14861 *GBA* noncarriers of PD (*GBA* − PD) were involved in the final meta-analysis, and 14 of them were either newly added publications or related data newly analyzed compared with the version published in 2015. Then, a series of symptoms containing depression, orthostatic hypotension, motor fluctuation, wearing-off, and freezing were newly analyzed due to more articles eligible. Besides, clinical features like family history, AAO, UPDRS-III, H-Y, and dementia previously analyzed were updated with new data added. Significant statistical differences were found in wearing-off, family history, AAO, UPDRS-III, and dementia (OR: 1.14, 1.65; MD: −3.61, 2.17; OR: 2.44; *p*: 0.03, <0.00001, <0.00001, 0.003, and <0.00001). Depression was slightly associated with *GBA* + PD (OR: 1.47; *p*: 0.04). Clinical symptoms such as H-Y, orthostatic hypotension, motor fluctuation, and freezing did not feature *GBA* + PD.

**Conclusion:**

Our results demonstrated that there were unique clinical features in *GBA* + PD which can help the management of the whole duration of PD patients.

## 1. Introduction

Parkinson's disease (PD), a common neurodegenerative disease, was featured by motor symptoms containing bradykinesia, resting tremor, rigidity, and postural instability. Nonmotor symptoms (NMS) such as cognitive impairment, olfactory dysfunction, and depression were also common in PD patients. Nowadays, the pathogenesis of PD remains elusive. Genetic factors have been demonstrated to cause PD and, to some extent, participate in modifying the phenotypes of PD [1–3].

*GBA* gene, encoding the lysosomal enzyme glucocerebrosidase (GCase), is the causative gene of Gaucher's disease (GD) [4]. *GBA* variants can increase the risk of PD up to 10 times, which was the strongest genetic factor contributing to the risk of PD [5, 6]. Nowadays, more than 300 mutations in *GBA* were reported [7–9]. The latest comprehensive meta-analysis had proved the importance of *GBA* variants such as L444P, N370S, R120W, IVS2 + 1G＞A, H255Q, D409H, RecNciI, E326K, and T369M to PD risks [10].

In addition to the contribution of *GBA* to the development of PD, studies have reported PD patients with *GBA* carriers (*GBA* + PD) manifested special clinical features compared to idiopathic PD. In the year of 2015, our group conducted a study combining the results of our new original research and meta-analysis on the association between *GBA* variants and the clinical features of PD [11]. The data indicated that *GBA* + PD are more inclined to onset at early age, initially with bradykinesia, have family history and develop to dementia when compared with *GBA* noncarriers of PD patients (*GBA* − PD). However, with more published articles, there were new clinical features such as depression, motor complications, and freezing gaits focused by researchers which will help draw a full picture of clinical features of *GBA* + PD or *GBA* − PD with complete motor symptoms (MSs) and NMSs [12–14]. Combined with newly published articles and newly involved data of previous articles, we performed a comprehensive analysis on clinical features of *GBA* + PD.

## 2. Methods

### 2.1. Selection Criteria

We conducted this meta-analysis based on PICOS (participants, interventions, controls, outcomes, and studies) rules.

#### 2.1.1. Participants

All PD patients being diagnosed with widely accepted diagnostic criteria [15].

#### 2.1.2. Interventions

DNAs were expanded by PCR-based methods or other accepted methods and analyzed by Sanger sequencing or other regular methods.

#### 2.1.3. Controls

Controls were PD patients without carrying *GBA* variants.

#### 2.1.4. Outcomes

A specific clinical feature of *GBA* carriers and noncarriers in PD patients were reported.

#### 2.1.5. Study Types

Original studies such as case-only study, cohort study, or case-control study were conducted.

### 2.2. Literature Search

We searched articles in English using Medline database in Pubmed, Embase database in Ovid, and the Cochrane database. Key words were “*GBA*,” “glucocerebrosidase,” “Parkinso^∗^” and “PD.” The latest search was done on March 1, 2018. Overlapping articles from different databases were excluded. Two researchers (Yuan Zhang and Li Shu) performed the search independently. In case of disagreements, a third researcher (Qiying Sun) was consulted to arrive at a consensus.

### 2.3. Data Extraction

Comprehensive data were retrieved including the following items: publication year, first author, ethnicity, country, number of *GBA* + PD and *GBA* − PD, and corresponding clinical information. Two researchers did the extraction independently. Another author was asked to participate in the process when confronted with problems. The quality of all case-control studies were assessed according to the Newcastle-Ottawa Scale (NOS) [16].

### 2.4. Statistical Analysis

The total statistical analysis was performed in Revman 5.3 software. The final results were demonstrated by pooled odds ratio (OR) or mean difference (MD) and 95% CI (confidence interval). When the data were dichotomous variables, pooled odds ratio (OR) was calculated, otherwise when the data were continuous outcomes, pooled mean difference (MD) was expressed. Heterogeneity was reflected by *Q* statistic (*p* value) and *I* ^2^ statistic. *p* > 0.1, *I* ^2^ ≤ 50% indicated that the heterogeneity was not significant and suggested a fixed model (FM) be applied. Otherwise, a random model (RM) was used. The shape of funnel plot was used to reflect publication biases. Sensitivity analysis was performed by removing each original study sequentially to test the stability of the results.

## 3. Results

**Figure 1:**
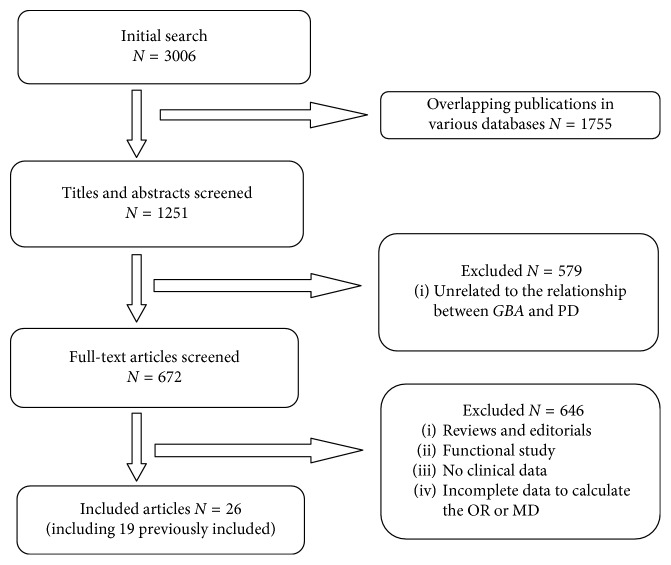
Flowchart illustrating the literature screening process.

The complete information of searching process is shown in the flowchart (Figure 1), and the information of all included studies is demonstrated in Table 1. A total of 26 articles including 931 *GBA* + PD and 14861 *GBA* − PD were involved in the final meta-analysis, and 14 of them, which contained 582 *GBA* + PD and 8217 *GBA* − PD, were either newly added publications or related data newly analyzed compared with the version published in 2015 [11]. Then, a series of symptoms containing depression, orthostatic hypotension, motor fluctuation, wearing-off, and freezing were newly analyzed due to more articles eligible. Besides, clinical features like family history, age at onset (AAO), Uniﬁed Parkinson's Disease Rating Scale Part III (UPDRS-III), Hoehn–Yahr (H-Y), and dementia previously analyzed were updated with new data added. Due to the importance of disease duration in clinical characteristics of disease, we first conducted comparison of disease duration between *GBA* + PD and *GBA* − PD. We found that there was no statistical difference between the two groups (MD: 0.17, *p*: 0.47) (Supplementary Table 1; Supplementary Figure 1).

For the five (depression, orthostatic hypotension, motor fluctuation, wearing-off, and freezing) newly involved clinical characteristics in this meta-analysis (Table 2, Supplementary Figure 1), they belonged to NMS and motor complications. As can be seen from Table 2, significant statistical difference was found in wearing-off (OR: 1.14; *p*: 0.03). Slightly statistical significance was found in depression of *GBA* + PD (OR: 1.47; *p*: 0.04). Clinical symptoms such as orthostatic hypotension, motor fluctuation, and freezing did not feature *GBA* + PD in this meta-analysis.

As to the five updated clinical features of *GBA* + PD with newly involved data (Table 2, Supplementary Figure 1), they were family history, AAO, UPDRS-III, H-Y, and dementia. From the tables, significant statistical differences were found in family history, AAO, UPDRS-III, and dementia (OR: 1.65; MD: −3.61, 2.17; OR: 2.44; *p*: <0.00001, <0.00001, 0.003, and <0.00001). We found a change in statistical differences in UPDRS-III scores from previous negative results, while we almost reached the same conclusion in analyzing family history, AAO, H-Y, and dementia.

All publications included were of high quality with the NOS scores above 7. According to the funnel plots (Supplementary Figure 2), the biases were rare. By removing articles one after another, the results of the remainder did not change significantly indicating that the results of our meta-analysis were stable.

## 4. Discussion

In our meta-analysis, we analyzed five new clinical features (depression, orthostatic hypotension, motor fluctuation, wearing-off, and freezing) and updated data of five previous analyzed clinical features (family history, AAO, UPDRS-III, H-Y, and dementia). We made the conclusion that *GBA* + PD patients had unique clinical features such as were more likely to have family history, earlier onset age, higher UPDRS-III scores, and develop dementia, depression, and wearing-off phenomena after adjusting disease duration.

Our meta-analysis about the demographic information of *GBA* + PD suggested that the carriers were more likely to have earlier age at onset with a mean of 3.6 years. Previous studies have shown that *GBA* + PD developed PD 1.7–6.0 years earlier than *GBA* − PD which were similar to our analysis [17]. Additionally, *GBA* + PD were more likely to have family history. These basic features of *GBA* carriers will contribute to the targeted screening of the gene in researches.

As to other clinical features such as MSs and NMSs, our analysis demonstrated severe MSs reflected by higher UPDRS-III scores accompanied by motor complications like wearing-off phenomena and high possibilities to develop NMSs such as dementia and depression. Previous studies [18–20] have suggested deteriorative manifestations of *GBA* + PD such as higher UPDRS-III scores, easily presenting dementia, and motor complications. Since separating different subcategories of PD is crucial to better understand disease mechanisms, predict disease progression, or design clinical researches, recently, Fereshtehnejad et al. [21] reported that important clinical features and scales such as UPDRS I-III scores, NMSs-related scales such as Montreal Cognitive Assessment (MoCA) evaluating cognitive functions, and Epworth Sleepiness Scale (ESS) evaluation sleep disturbances were key factors defining clinical subtypes of PD. Our analysis found unique clinical features of *GBA* + PD which almost matched a diffuse malignant subtype in PD in the previous classifications which needed a more active treatment strategy for the deleterious prognosis.

The mechanism underlying *GBA* + PD prominent clinical features remains elusive. Some studies suggested that *GBA* mutations can cause dysfunctional GCase which finally led to *α*-synuclein aggregations in PD brains and in dopaminergic neurons [22]. As *α*-synuclein was vital pathological feature in PD brains, the promotion of *GBA* mutations to *α*-synuclein aggregations may explain the deleterious clinical features of *GBA* + PD. The dysfunctions in pathways outside classic basal ganglia may explain the NMS features of *GBA* + PD. The cortex dysfunction caused by global brain degeneration can damage functions of specific areas of brains and cause dementia or depression [23]. However, the number of researches was limited, and more mechanism studies were needed in the future.

Previously, our comprehensive meta-analysis in *GBA* variants had proved the importance of *GBA* mutations to PD risks [10]. To clarify the role of *GBA* in PD clinical features more clearly, we did this meta-analysis. Our results demonstrated a clear phenotype-genotype correlation in *GBA* *+* PD. Knowing the unique features of *GBA* carriers will contribute to predicting the clinical course of *GBA* + PD and be benefit for the symptomatic treatments. The results of this meta-analysis can do a contribution to the precise treatments based on genetic screening and help delay the progression of the disease with more active and effective therapeutic strategies.

To evaluate the meta-analysis more objectively, there were some limitations which cannot be ignored. First, possible biases were inevitable because the included original studies were cross-sectional and possible biases existed in pooled analyses of these studies such as age, gender, or other correlated clinical phenotypes. Further longitudinal designed studies will be needed to confirm these results. Second, because most of these researches included mixed different specific variants together as *GBA* + PD or *GBA* − PD (Supplementary Table 1), we could not separate each variant with corresponding phenotype data and were not able to conduct pooled analysis based on specific variants of *GBA*. With more original articles conducted based on specific variants of *GBA* and phenotype, we may be able to do a more accurate analysis to help understand the relationship between the genotype and phenotype better. Third, although our updated meta-analysis was a comprehensive pooled analysis of *GBA*-associated clinical presentations, for the limited articles, we failed to prove the relationship of other clinical features such as rapid eye movement sleep behavior disorder (RBD) or freezing which were demonstrated to be associated with *GBA* previously [24].

## 5. Conclusion

Our meta-analysis suggested an increased risk of having family history, dementia, depression, wearing-off, earlier onset age, and higher UPDRS-III scores with *GBA* + PD. However, variants in *GBA* had no relationship with H-Y, orthostatic hypotension, motor fluctuations, and freezing in PD. More data were needed to do complete analysis on different variants and different ethnics of *GBA*, and the corresponding clinical manifestations which can help the management of the whole duration of PD patients.

## Figures and Tables

**Table 1:** The characteristics of the related phenotypes data updated in all publications included.

Year, first author	NOS	Country	Groups	*n*	Family history	AAO	UPDRS-III	H-Y	Dementia	Depression	Orthostatic hypotension	Wearing-off	Motor fluctuation	Freezing
2017, Cilia et.al. [25]^*∗*^	8	Italy	*GBA* + PD	123	38	52.4 ± 10.2	33.5 ± 14.2	—	25 (93)	—	12 (65)	—	48 (82)	28 (92)
			*GBA* − PD	2641	446	57.4 ± 10.6	30.4 ± 13.7	—	240 (1254)	—	89 (840)	—	726 (168)	332 (1210)
2017, Davis et.al. [26]^*∗*^	7	American	*GBA* + PD	27	—	62.0 ± 9.0	31.8 ± 10.6	—	9	—	—	—	—	—
			*GBA* − PD	675	—	68.4 ± 8.6	27.5 ± 12.9	—	58	—	—	—	—	—
2016, Thaler et al. [27]^*∗*^	7	Israel	*GBA* + PD	12	—	51.4 ± 10.7	28.8 ± 9.6	2.9 ± 0.6	—	—	—	—	—	—
			*GBA* − PD	12	—	58.7 ± 5.7	21.7 ± 6.5	2.1 ± 0.7	—	—	—	—	—	—
2016, Swan et al. [28]^*∗*^	7	Israel	*GBA* + PD	31	—	57.0 ± 12.7	16.7 ± 8.7	2.3 ± 1.1	—	10	—	—	—	—
			*GBA* − PD	55	—	59.7 ± 11.4	20.4 ± 13.2	2.2 ± 0.9	—	7	—	—	—	—
2016, Dan et al. [29]^*∗*^	8	China	*GBA* + PD	40	—	—	—	—	—	16	—	—	—	—
			*GBA* − PD	1007	—	—	—	—	—	191	—	—	—	—
2015, Gan-Or et al. [24]^*∗*^	7	Israel	*GBA* + PD	19	—	—	—	—	—	—	—	—	—	—
			*GBA* − PD	101	—	—	—	—	—	—	—	—	—	—
2014, Brockmann et al. [22]^*∗*^	7	Germany	*GBA* + PD	33	—	—	—	2.7 ± 0.7	—	—	—	—	—	—
			*GBA* − PD	26	—	—	—	2.5 ± 0.7	—	—	—	—	—	—
2014, Wang et al. [30]^#^	8	China	*GBA* + PD	49	—	—	—	—	—	28 (49)	9 (34)	11 (37)	5 (37)	—
			*GBA* − PD	1366	—	—	—	—	—	583 (1366)	221 (843)	169 (922)	90 (924)	—
2014, Malec-Litwinowicz et.al. [31]^#^	7	Poland	*GBA* + PD	5	—	—	—	—	—	4	1	—	—	—
			*GBA* − PD	117	—	—	—	—	—	43	21	—	—	—
2014, Li et al. [32]^#^	7	Japan	*GBA* + PD	34	—	—	—	—	—	—	5	20	—	—
			*GBA* − PD	113	—	—	—	—	—	—	21	49	—	—
2014, Pulkes et al. [33]^#^	7	China, Thailand	*GBA* + PD	17	—	—	—	—	—	—	—	8	—	2
			*GBA* − PD	191	—	—	—	—	—	—	—	82	—	21
2013, Kumar et al. [34]^#^	7	Serbia	*GBA* + PD	21	—	—	—	—	—	—	—	—	—	0 (19)
			*GBA* − PD	339	—	—	—	—	—	—	—	—	—	8 (287)
2011, Lesage et al. [35]^#^	8	Europeans	*GBA* + PD	100	—	—	—	—	—	—	—	—	47 (76)	—
			*GBA* − PD	1291	—	—	—	—	—	—	—	—	532 (902)	—
2008, Gan-Or et al. [36]^#^	7	Israel	*GBA* + PD	71	—	—	—	—	—	6	—	—	—	—
			*GBA* − PD	283	—	—	—	—	—	28 (280)	—	—	—	—

Abbreviations: ^*∗*^publications newly updated; ^#^publications previously included, and this table only exhibits the updated clinical data in the previous publications included; other clinical features were shown in the published manuscript [11]. PD, Parkinson's disease; *GBA* + PD, PD with *GBA* mutations; *GBA* − PD, PD without *GBA* mutations; AAO, age at onset; UPSRS-III, the Part III of Unified Parkinson Disease Rating Scale; H-Y, Hoehn–Yahr Rating Scale. AAO, UPDRS-III, and H-Y were presented as mean and standard deviation; others were shown as count data. *n*, total number of patients whose clinical information was available in each group.

**Table 2:** *GBA*-related phenotypes updated to our previous meta-analysis.

Phenotypes	Number of articles (total/updated)	Total number of *GBA* + PD	Total number of *GBA* − PD	OR or MD (95% CI) updated	*p* value	Previous OR or MD (95% CI)
Family history^*∗*^^a^	11/1	558	9330	**1.65 (1.34, 2.02)**	**<0.00001**	**1.5 (1.18, 1.91)**
AAO^*∗*^^b^	17/4	622	11079	**−3.61 (−5.04, −2.17)**	**<0.00001**	**−3.10 (−4.88, −1.32)**
UPDRS-III^*∗*^^b^	9/4	335	6100	**2.17 (0.72, 3.62)**	**0.003**	1.61 (−0.65, 3.87)
H-Y^*∗*^^b^	11/3	275	3863	**0.18 (0.00, 0.35)**	**0.05**	0.06 (−0.06, 0.17)
Dementia^*∗*^^a^	8/2	224	2696	**2.44 (1.79, 3.33)**	**<0.00001**	**3.21 (1.97, 5.24)**
Depression^#a^	5	196	2825	**1.47 (1.02, 2.13)**	**0.04**	**—**
Orthostatic hypotension^#a^	4	138	1913	1.24 (0.79, 1.94)	0.35	**—**
Motor fluctuation^#a^	3	195	2894	0.9 (0.66, 1.24)	0.53	**—**
Wearing-off^#a^	3	88	1226	**1.68 (1.05, 2.69)**	**0.03**	**—**
Freezing^#a^	3	128	1688	1.14 (0.74, 1.77)	0.55	**—**

Abbreviations: ^*∗*^phenotypes updated new publications; ^#^phenotypes newly analyzed. PD, Parkinson's disease; *GBA* + PD, PD with *GBA* mutations; *GBA* - PD, PD without *GBA* mutations; AAO, age at onset; UPSRS-III, the Part III of Unified Parkinson Disease Rating Scale; OR, odds ratio; MD, mean deviation; CI, confidence interval. Bold OR or MD, 95% CI, and *p* values reflected statistically significance results; a, dichotomous variables reflected by OR (95% CI); b, continuous outcomes reflected by MD (95% CI).

## Supplementary Materials

Supplementary Materials Supplementary Figure 1: forest plots of the association between phenotypes and PD risks in total. (A)–(J) respond to the phenotypes of family history, age at onset, UPDRS-III, H-Y, dementia, depression, orthostatic hypotension, motor fluctuation, wearing-off, and freezing individually. Supplementary Figure 2: funnel plots of the association between phenotypes and PD risks in total. (A)–(J) respond to the phenotypes of family history, age at onset, UPDRS-III, H-Y, dementia, depression, orthostatic hypotension, motor fluctuation, wearing-off, and freezing individually. Supplementary Table 1: the GBA variants reported in included articles. Abbreviations: C, carriers of GBA variants; NC, noncarriers of GBA variants; NA, not available.
