# Supplementary material for: A Meta-Analysis of GBA-Related Clinical Symptoms in Parkinson's Disease
Source: Parkinsons Dis. 2018 Sep 27;2018:3136415. doi: 10.1155/2018/3136415 (PMC6180987; doi:10.1155/2018/3136415)
Supplement: Supplementary Materials — Supplementary Figure 1: forest plots of the association between phenotypes and PD risks in total. (A)–(J) respond to the phenotypes of family history, age at onset, UPDRS-III, H-Y, dementia, depression, orthostatic hypotension, motor fluctuation, wearing-off, and freezing individually. Supplementary Figure 2: funnel plots of the association between phenotypes and PD risks in total. (A)–(J) respond to the phenotypes of family history, age at onset, UPDRS-III, H-Y, dementia, depression, orthostatic hypotension, motor fluctuation, wearing-off, and freezing individually. Supplementary Table 1: the GBA variants reported in included articles. Abbreviations: C, carriers of GBA variants; NC, noncarriers of GBA variants; NA, not available. [file 3136415.f1.docx]

A meta-analysis of *GBA* related clinical symptoms in Parkinson’s disease

**Yuan Zhang^2 †^, Li Shu^2 †^, Xun Zhou^2^, Hongxu Pan^2^, Qian Xu^2, 3,4^, Jifeng Guo^2,3,4,6,7,8^, Beisha Tang^1, 2,3,4,5,6,7,8^, Qiying Sun^1,3,4*^**

**^†^** These authors contributed equally to this work and are co-first authors.

^*^ Correspondence: Qiying Sun [sunqiying2015@163.com](mailto:sunqiying2015@163.com)


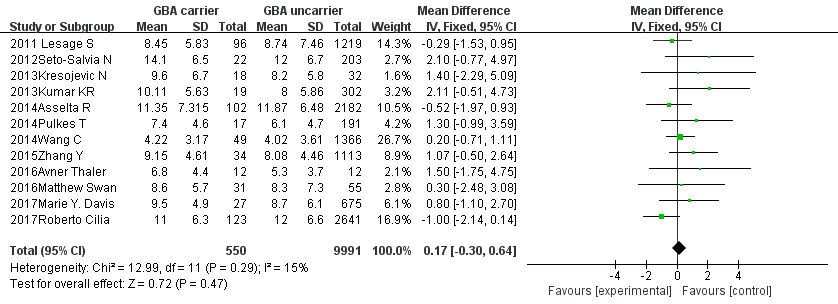


1. Forest plot of the association between duration and PD risks in total.


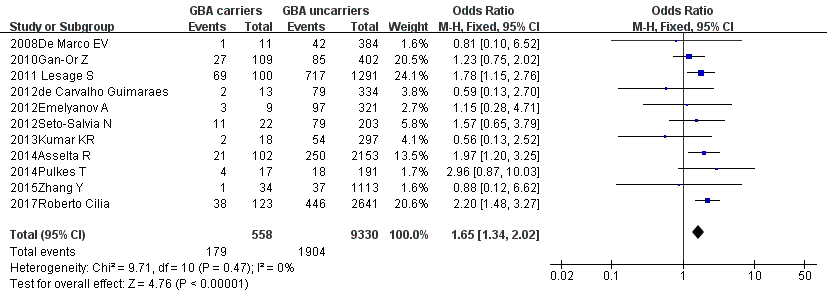


1. Forest plot of the association between family history and PD risks in total.


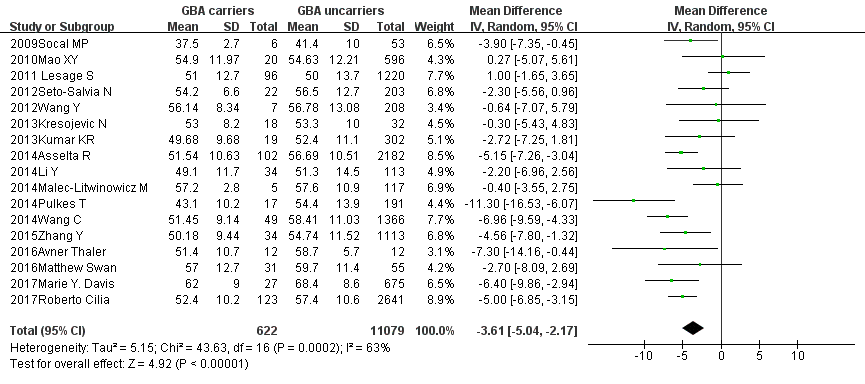


1. Forest plot of the association between age at onset and PD risks in total.


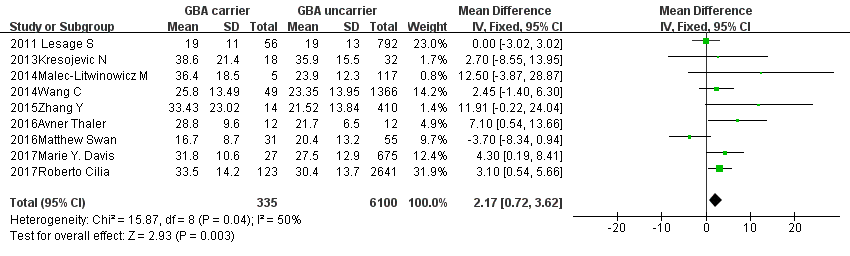


1. Forest plot of the association between UPDRS-III and PD risks in total.


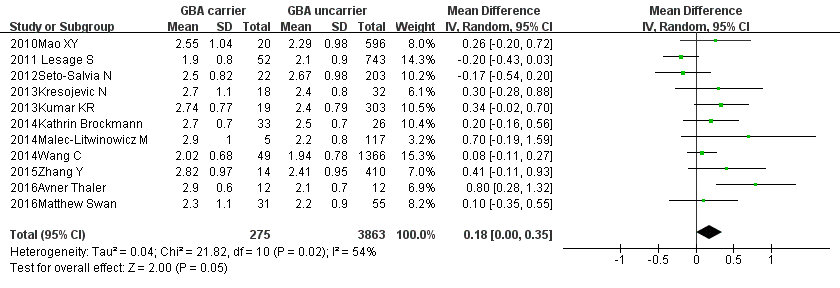


1. Forest plot of the association between H-Y and PD risks in total.


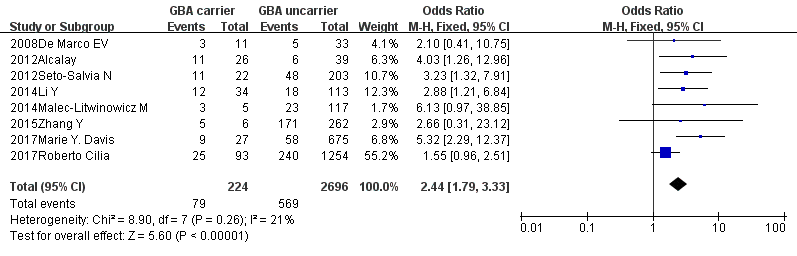


1. Forest plot of the association between dementia and PD risks in total.


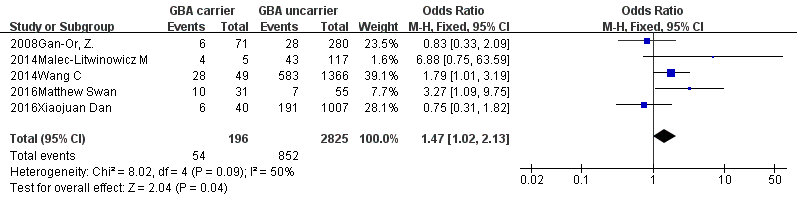


1. Forest plot of the association between depression and PD risks in total.


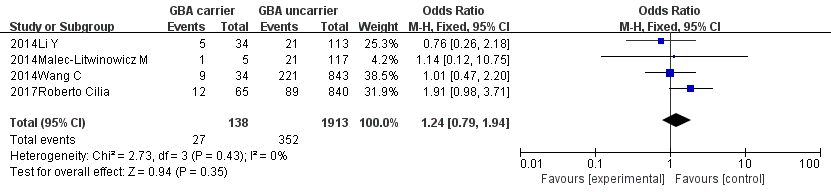


1. Forest plot of the association between orthostatic hypotension and PD risks in total.


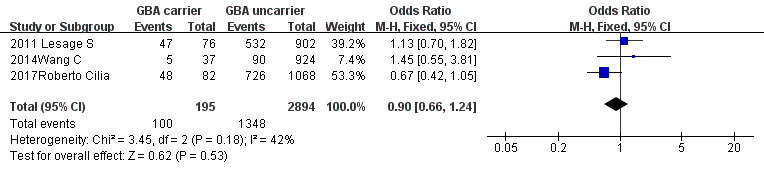


1. Forest plot of the association between motor fluctuation and PD risks in total.


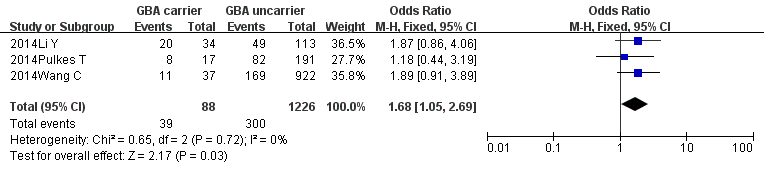


1. Forest plot of the association between wearing-off and PD risks in total.


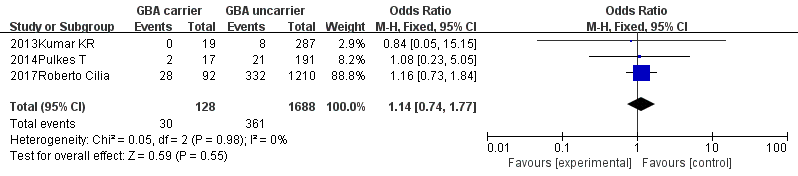


1. Forest plot of the association between freezing and PD risks in total.

**Supplementary Figure 1**: Forest plots of the association between phenotypes and PD risks in total. (A)-(K) respond to the phenotypes of duration, family history, age at onset, UPDRS-III, H-Y, dementia, depression, orthostatic hypotension, motor fluctuation, wearing-off and freezing individually.


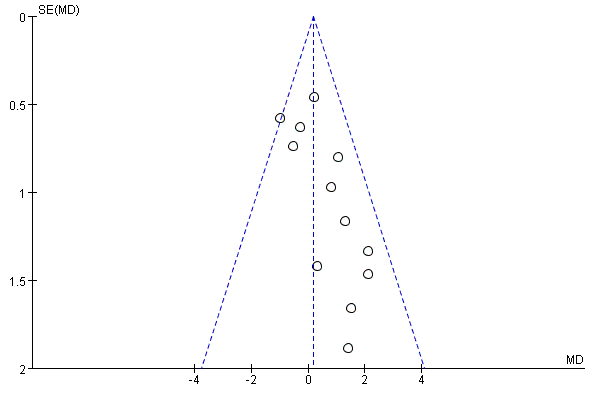


1. Funnel plot of the association between duration and PD risks in total.


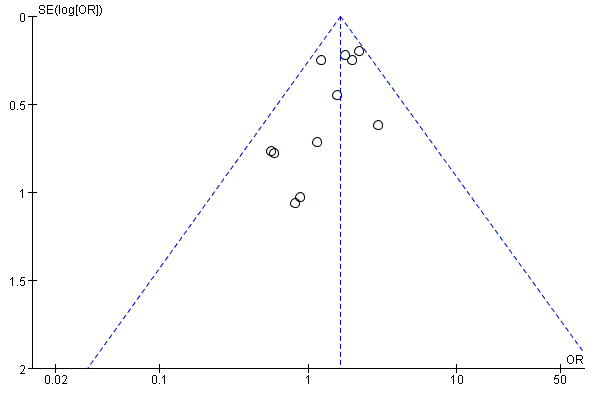


1. Funnel plot of the association between family history and PD risks in total.


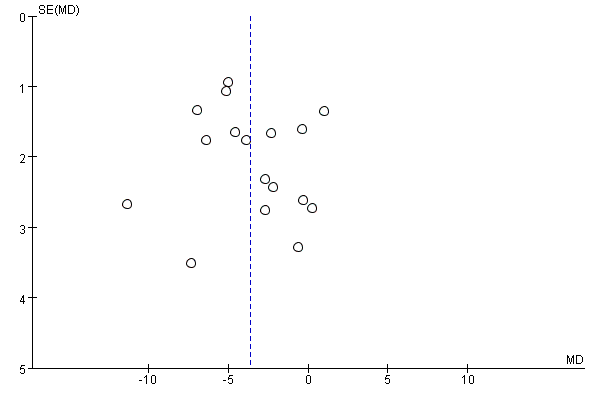


1. Funnel plot of the association between age at onset and PD risks in total.


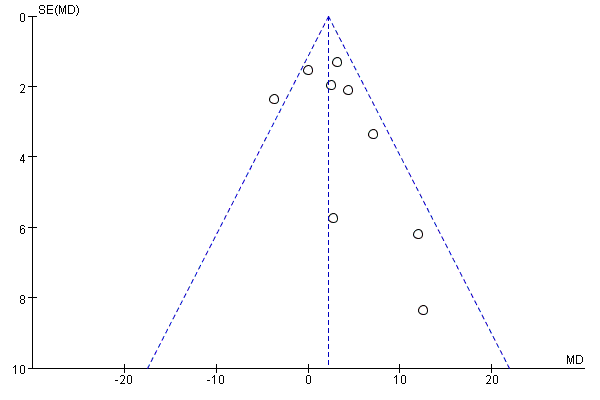


1. Funnel plot of the association between UPDRS-III and PD risks in total.


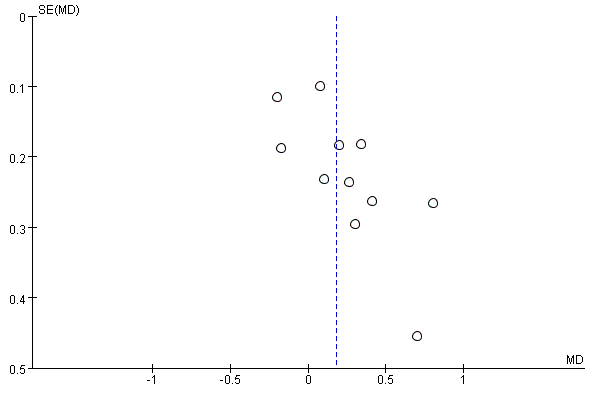


1. Funnel plot of the association between H-Y and PD risks in total.


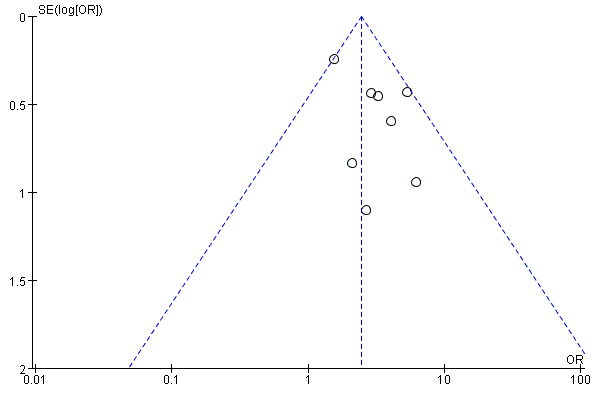


1. Funnel plot of the association between dementia and PD risks in total.


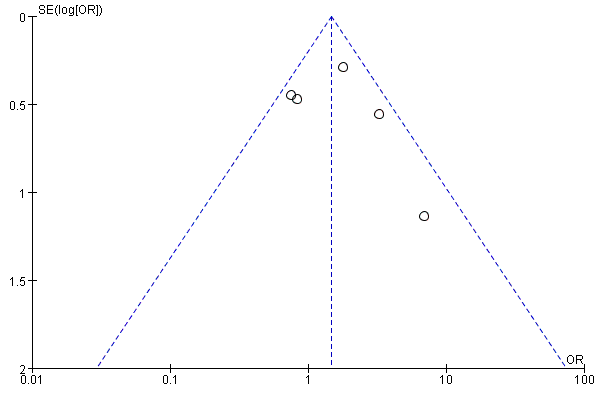


1. Funnel plot of the association between depression and PD risks in total.


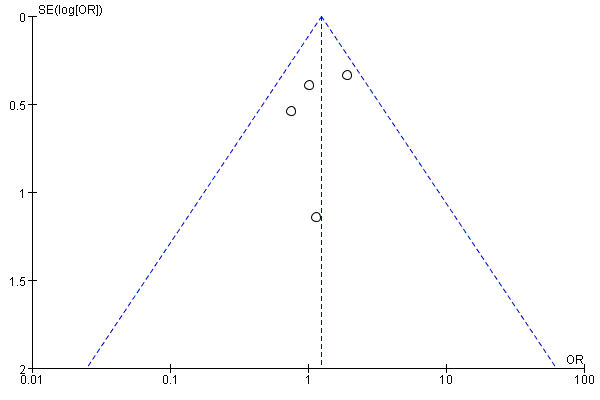


1. Funnel plot of the association between orthostatic hypotension and PD risks in total.


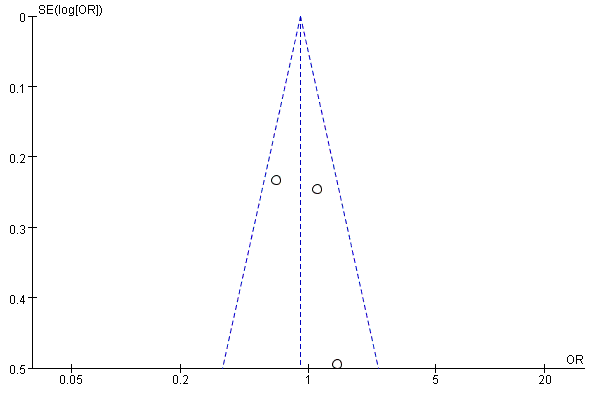


1. Funnel plot of the association between motor fluctuation and PD risks in total.


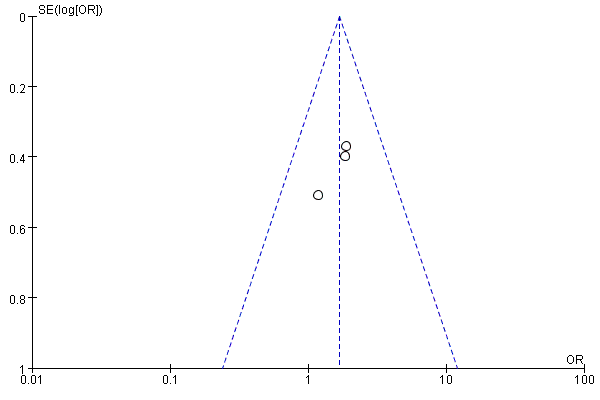


1. Funnel plot of the association between wearing-off and PD risks in total.


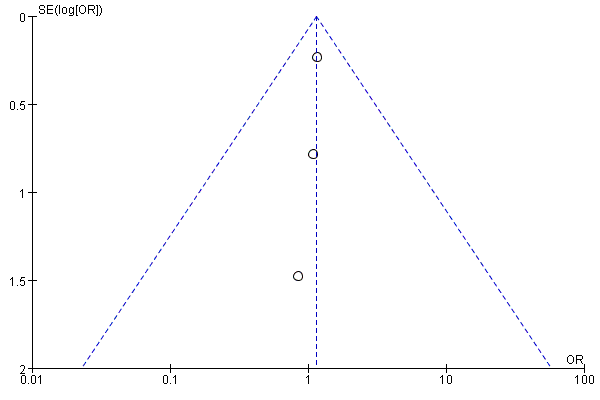


1. Funnel plot of the association between freezing and PD risks in total.

**Supplementary Figure 2**: Funnel plots of the association between phenotypes and PD risks in total. (A)-(K) respond to the phenotypes of duration, family history, age at onset, UPDRS-III, H-Y, dementia, depression, orthostatic hypotension, motor fluctuation, wearing-off and freezing individually.

Supplementary Table 1. The *GBA* variants reported in included articles.

| Year and First author | Country | Disease duration(C/NC) | Variants |
| --- | --- | --- | --- |
| *2017Roberto Cilia | Italy | 11.0±6.3/12.0±6.6 | N370S, L444P |
| *2017Marie Y. Davis | American | 9.5±4.9/8.7±6.1 | E326K |
| *2016Avner Thale | Israel | 6.8±4.4/5.3±3.7 | N370S, L444P, c.84insG, IVS2+1G->A, V394L, R496H, RecTL |
| *2016Matthew Swan | Israel | 8.6±5.7/8.3±7.3 | N370S, 84GG, R496H, L444P |
| *2016Xiaojuan Dan | China | NA | L444P |
| *2015Ziv Gan-Or | Israel | NA | NA |
| *2014Kathrin Brockmann | Germany | NA | N370S, L444P |
| #2014Wang C | China | 4.22±3.17/4.02±3.61 | L444P |
| #2014Malec-Litwinowicz M | Poland | NA | N370S, T369M |
| #2014Li Y | Japan | NA | R120W, D409H, L444P, indel, RecNciI, G64V, W393X, I489V |
| #2014Pulkes T | China, Thailand | 7.4±4.6/6.1±4.7 | L444P, IVS2+1G->A, N386K, P428S, IVS9+3G>C, IVS10-9_10GT>AG, c.1309delG |
| #2013Kumar KR | Serbia | 10.11±5.63/8.0±5.86 | N370S, D409H H255Q, L444P, R463C, RecNciI |
| #2011Lesage S | Europeans | 8.45±5.83/8.74±7.46 | N370S, L444P |
| #2008Gan-Or, Z | Israel | NA | N370S |
| 2015Zhang | China | 9.15±4.61/8.08±4.46 | L444P |
| 2014Asselta | Italy | 11.35±7.315/11.87±6.48 | N370S, L444P, D443N, IVS10+1G>T |
| 2013Kresojevic | Serbia | 9.6±6.7/8.2±5.8 | D409H, N370S, L444P, D380V, E388K |
| 2012Wang | China | NA | L444P |
| 2012Seto-Salvia | Europe | 14.1±6.5/12.0±6.7 | N370S, L444P, L144V, S488T, M123T, G202R, I260T, T369M, W393R, D409H, RecNciI |
| 2012Emelyanov | Russia | NA | N370S, L444P |
| 2012de Carvalho Guimaraes | Brazil | NA | N370S, L444P |
| 2012Alcalay | Multi-sites | NA | N370S, L444P |
| 2011Huang | China | NA | L444P, RecNciI, D409H |
| 2010Mao | China | NA | L444P |
| 2009Socal | Brazil | NA | N370S, L444P |
| 2008De Marco | Italy | NA | N370S, L444P |

Abbreviations: C, carriers of *GBA* variants; NC, non-carriers of *GBA* variants; NA, not available.
